# Unraveling the Metabolic Changes in Acute Pancreatitis: A Metabolomics-Based Approach for Etiological Differentiation and Acute Biomarker Discovery

**DOI:** 10.3390/biom13101558

**Published:** 2023-10-22

**Authors:** Greta Dancu, Cristi Tarta, Carmen Socaciu, Felix Bende, Mirela Danila, Roxana Sirli, Ioan Sporea, Bogdan Miutescu, Alina Popescu

**Affiliations:** 1 Center for Advanced Research in Gastroenterology and Hepatology, Department of Internal Medicine II, Division of Gastroenterology and Hepatology, “Victor Babes” University of Medicine and Pharmacy Timisoara, Eftimie Murgu Sq. No. 2, 300041 Timisoara, Romaniadanila.mirela@umft.ro (M.D.); sirli.roxana@umft.ro (R.S.); popescu.alina@umft.ro (A.P.); 2 Department X, 2nd Surgical Clinic, Researching Future Chirurgie 2, “Victor Babes” University of Medicine and Pharmacy Timisoara, Eftimie Murgu Sq. No. 2, 300041 Timisoara, Romania; 3 Department of Food Science, Faculty of Food Science and Technology, University of Agricultural Sciences and Veterinary Medicine Cluj-Napoca, 400372 Cluj-Napoca, Romania; carmen.socaciu@usamvcluj.ro

**Keywords:** acute pancreatitis, metabolomics, lipidomics, glycerophospholipids, glycerolipids, fatty acyls, sterol lipids, biomarkers

## Abstract

Acute pancreatitis (AP) remains a challenging medical condition, where a deeper metabolic insight could pave the way for innovative treatments. This research harnessed serum metabolomics to discern potential diagnostic markers for AP and distinguish between its biliary (BAP) and alcohol-induced (AAP) forms. Leveraging high-performance liquid chromatography coupled with mass spectrometry, the metabolic signatures of 34 AP patients were contrasted against 26 healthy participants, and then between different etiologies of AP. The results identified metabolites primarily from glycerophospholipids, glycerolipids, fatty acyls, sterol lipids, and pteridines and derivative classes, with the Human Metabolome Database aiding in classification. Notably, these metabolites differentiated AP from healthy states with high AUROC values above 0.8. Another set of metabolites revealed differences between BAP and AAP, but these results were not as marked as the former. This lipidomic analysis provides an introduction to the metabolic landscape of acute pancreatitis, revealing changes in multiple lipid classes and metabolites and identifying these metabolites. Future research could add and discover new diagnostic biomarkers and therapeutic strategies enhancing the management of acute pancreatitis.

## 1. Introduction

Acute pancreatitis (AP) is a prevalent and severe gastrointestinal disorder that globally affects between 5 and 80 out of 100,000 subjects, with an observed increase in incidence over the years [1,2]. Described as an autodigestive disease, AP results from the inflammation of the exocrine pancreas, responsible for secreting digestive enzymes into the duodenum. The pathogenesis of AP remains largely elusive, with premature intracellular protease activation and oxidative stress among the suggested mechanisms underpinning disease onset [3]. Once activated, these intracellular proteases induce acinar cell injury, causing cell membrane disruption, edema, interstitial hemorrhaging, and necrosis and igniting an inflammatory response marked by leukocyte infiltration [4].

The most prevalent etiologies of AP to date are alcohol and gallstones, reported a decade ago to account for around 20% and 40–50% of cases, respectively, with geographic differences [5,6]. Different types of AP demand divergent treatment strategies; for instance, patients with obstructive biliary stones necessitate stone removal. Consequently, there is an increasing need for rapid and accurate methods to diagnose AP by its specific pathogenesis [7,8]. The specificity and accuracy of the standard diagnostic criteria of increased serum amylase and lipase enzymes fall short of being absolute [9,10,11].

Emerging from the “omics” frontier, metabolomics—a component of systems biology—offers promising insights into the diagnosis and pathogenesis of various diseases, including AP. Metabolomics facilitates the comprehensive profiling of small endogenous metabolites present in blood or urine, providing a robust platform for mapping disease-specific perturbations in biochemical pathways [12,13,14]. This potential capability to identify predictive biomarkers associated with disease stages, severity, or etiology recommends metabolomics as a powerful tool in the diagnosis of AP. Preliminary evidence suggests that metabolite profiling could serve not only as a diagnostic instrument for AP but also as a means of phenotypic detection and pharmaceutical innovation [15].

A better understanding of AP’s pathologic mechanism at the metabolic level is a precursor to novel drug discovery. Metabolomics, with its capacity to offer specific and sensitive biomarkers, could elucidate the potential associations between metabolites and physiological or pathological changes, thus distinguishing different states of the biological system [15,16]. As the final products of cellular regulatory processes, metabolite levels are considered the definitive response of the biological system to genetic or environmental alterations [1].

The present study aims to harness the power of serum metabolomics to identify potential diagnostic biomarkers for AP compared to healthy subjects and to differentiate between its two major forms—biliary AP (BAP) and alcohol-induced AP (AAP).

## 2. Materials and Methods

### 2.1. Study Period and Participants

This study was a prospective observational cohort study, conducted at a tertiary department of gastroenterology, and was performed in compliance with relevant guidelines and regulations. Ethical approval for this study was obtained from the Emergency County Timisoara Hospital Committee for Ethics (decision number 206, 7 September 2020). All patients provided written informed consent.

#### 2.1.1. Study Participants

Patients admitted with acute pancreatitis (AP) between 1 October 2020 and 31 November 2020 were considered for inclusion. AP was confirmed when at least two of the following criteria were met: consistent abdominal pain, serum lipase level 3-fold higher than the normal level, or typical aspects of AP on a CT scan. These patients were allocated to the P group of our study.

Patients were excluded if they were concurrently diagnosed with SARS-CoV-2 infection, referred from other hospitals late after the onset of the disease, were under 18 years old, or were pregnant. A control group comprising hospital employees without known gastrointestinal disease and matched for age and sex distribution, who agreed to participate in this study, was also recruited and allocated to the C (control) group.

#### 2.1.2. Data Collection

Patients’ demographic and clinical data were collected at admission and included age, gender, vital signs, complete blood count, serum chemistry, and disease severity scores (BISAP and Ranson).

The Bedside Index for Severity in Acute Pancreatitis (BISAP) score, comprising variables like blood urea nitrogen (BUN) level, impaired mental status, systemic inflammatory response syndrome (SIRS), age > 60 years, and the presence of pleural effusion, was assessed at admission, utilizing the most severe parameters within the first 24 h [17].

The Ranson score was also calculated with five admission parameters (age, white blood cell count, blood glucose, AST, LDH) and six parameters assessed 48 h post-admission (hematocrit, increase in BUN, serum calcium, arterial PO_2_, base deficit, and fluid sequestration) [18].

In addition, we gathered data on etiology (biliary, named BAP; alcohol-induced, named AAP; and non-biliary–non-alcoholic), disease severity (categorized as mild (MAP), moderate (MSAP), or severe AP (SAP) according to the Revised Atlanta Criteria), hospital stay duration, and patient mortality [19].

Alcohol consumption assessment was based on self-reported consumption, using the AUDIT C questionnaire, where for females, scores ≥ 3 are consistent with alcohol misuse, and for males, scores ≥ 4 are consistent with alcohol misuse [20]. Binge drinking was defined using the criteria of the National Institute on Alcoholism and Alcohol Abuse (NIAAA), where for a typical adult, this pattern corresponds to consuming 5 or more drinks (male), or 4 or more drinks (female), in about 2 h [21].

Follow-up was performed 90 days after admission by clinic visit or by phone.

### 2.2. Blood Collection and Processing

The blood samples were collected on the second day of admission. Blood serum samples were collected according to standardized procedures in accordance with the ethical standards of the institutional and national research ethical committee and with the 1964 Helsinki Declaration and its later amendments for ethical standards.

The blood was collected in vacutainer tubes without anticoagulant, kept at room temperature for 30 min to allow clotting, and centrifuged for 10 min at 3000 rpm (4 °C) to separate clear serum. After separation, the blood serum was stored at −80 °C. To a volume of 0.3 mL serum, 0.7 mL from a mixture of methanol and acetonitrile (1:1) was added to precipitate proteins. The mixture was vortexed for 1 min and kept at −20 °C overnight; after defreezing, it was vortexed again for 1 min. After mixing, the vials were centrifuged at 12,500 rpm (4 °C) for 10 min, and the supernatant was collected and filtered through PTFE filters of 0.25 µm. All samples were processed in duplicate. Several QC samples obtained from each group were used in parallel in order to calibrate the HPLC separations.

HPLC–QTOF-ESI + MS analysis of blood serum: Aliquots of 3 µL serum were subjected to ultrahigh-pressure chromatography on a Thermo Scientific HPLC UltiMate 3000 system equipped with a DionexUltiMate 3000 quaternary pump system (UHPLC), a DionexUltimate 3000 photodiode array detector, a column oven, and an autosampler. Serum metabolites were separated using a Thermo Scientific (Waltham, MA, USA) C18 reverse-phase column (Acquity, UPLC C18 BEH, Dionex, Sunnyvale, CA, USA) (5 µm, 2.1 × 75 mm) at 25 °C and a flow rate of 0.3 mL/min. The mobile phase was represented by a gradient of eluent A (water containing 0.1% formic acid) and eluent B (methanol–acetonitrile, 1:1, containing 0.1% formic acid). The gradient system consisted of 97% A (min 0), 93% A (min 3), 75% A (min 6), 50% A (min 8), 15% A (min 10), and 97%A (min 15), followed by 3 min with 97% A. The total running time was 18 min. The mass spectrometry was performed on a Bruker Daltonics MaXis Impact QTOF instrument operating in positive ion mode (ESI+). The mass range was set between 50 and 1000 m/z. For measurements, the nebulizing gas pressure was set at 2.8 bar, the drying gas flow at 12 L/min, and the drying gas temperature at 300 °C. Before each chromatographic run, a calibrant solution of sodium formate was injected. The control of the instrument, acquisition, and data processing were performed using Chromeleon, TofControl 3.2, Hystar 3.2, and Data Analysis 4.2 (Bruker Daltonics, Billerica, MA, USA).

### 2.3. Statistical Analysis

Standard laboratory data were analyzed using MedCalc software(version 19.3). Continuous variables were expressed as mean ± standard deviation (SD) and categorical variables as percentages.

To compare the mean values between the two independent groups, the independent samples *t*-test (or Welch’s *t*-test, as appropriate) was applied; the chi-square test of independence was used to compare proportions. A two-tailed *p*-value of less than 0.05 was considered indicative of statistical significance.

The Bruker software Data Analysis 4.2 attached to the instrument was used to process the acquired data, after 3 replications for each sample. The mean m/z vs. peak intensity values were considered for each sample. First, from the base peak chromatogram (BPC), using the Dissect or the Find Molecular Features (FMF) algorithm, an advanced bucket matrix was generated. The matrix released by Data Analysis contained the retention time, the peak areas and intensities, and the signal/noise (S/N) ratio for each component together with its m/z value. The mass spectra were also recorded. Generally, the number of separated compounds ranged between 350 and 400.

In this first step, a matrix for all samples was obtained and saved as an Excel file. To eliminate the small signals with S/N values under 5, a first filtration was performed and then a second matrix containing m/z values and peak intensities was saved and filtered in a second step eliminating the small intensities, <1000. The number of peaks remained at 180–200. Only metabolites that were detected in more than 60% of the samples were included in the statistical analysis, so to produce an adequate alignment of the peak’s m/z values, the online software from bioinformatica.isa.cnr.it/NEAPOLIS was applied. The aligned matrix (3) allowed the calculation of mean intensity values and standard deviation for the control group and for group P. The aligned matrix (containing 120 m/z values) as a .csv file was uploaded to the specialized online platform Metaboanalyst 5.0. (https://www.metaboanalyst.ca/MetaboAnalyst/; accessed on 20 March 2021). The statistical analysis included principal component analysis (PCA) and partial least squares discriminant analysis (PLSDA) combined with VIP ranking and cross-validation. On the same platform, the random forest algorithm was applied for a prediction of molecules ranked by their contributions to classification accuracy (mean decrease accuracy). Finally, using biomarker analysis, the receiver operating characteristic (ROC) curves were obtained and the values of areas under ROC curves (AUROCs) were determined. Therefore, the molecules identified were ranked according to their sensitivity/specificity. AUROC values higher than 0.800 were considered as significant. Finally, the enrichment analysis allowed the identification of specific classes of lipids affected in acute pancreatitis compared to controls.

The identification of molecules that can be considered potential biomarkers was performed considering the precursor values obtained (m/z), confronted with the LC-MS search considering a max error of 1 ppm. The two most relevant databases for the identification were LIPID MAPS^®^ Lipidomics Gateway and Human Metabolome Database (https://hmdb.ca/; accessed on 22 March 2021). Preliminarily, in our lab, we checked the m/z values with some pure standards for confirmation.

## 3. Results

During the study period, a total of forty-five patients diagnosed with acute pancreatitis (AP) were initially considered for inclusion in this study. Following the application of exclusion criteria, eleven patients were excluded from this study. Among the excluded patients, six were diagnosed with a concomitant SARS-CoV-2 infection, three patients presented late after the onset of the disease, and two patients were under 18 years old.

Consequently, a total of thirty-four patients with AP were finally included in this study and constituted the patient group (group P). The P group consisted of 52% (18/34) males, with an average age of 57 ± 16 years old.

A control group (group C) was also included in this study, comprising twenty-six healthy individuals with no known gastrointestinal diseases. The control group was made up of 53% (14/26) males, with a mean age of 54 ± 6 years old.

The detailed characteristics of the patients are presented in Table 1 and Table 2.

The length of the hospital stay was 6 ± 3 days. The total mortality rate was 5%. Etiology was BAP 61.75% (21/34), AAP 20.60% (7/34), and non-BAP/AAP 17.65% (6/34). Severity of AP was classified as follows: MAP 41% (14/34), MSAP 52% (18/34), and SAP 5% (2/34).

### 3.1. Multivariate Analysis to Discriminate between Acute Pancreatitis and Control Group

#### 3.1.1. PLSDA Analysis and VIP Scores

**Figure 1:**
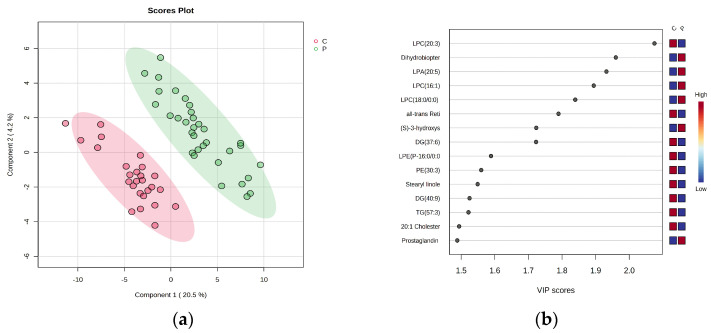
(**a**) PLSDA score plot, showing the discrimination between C and P groups. (**b**) Ranking of VIP scores for the first 15 molecules which may explain the discrimination between groups C and P.

Statistical analysis considered 120 m/z values, and the number of identified molecules was 69 (see Supplementary File Table S1). The unidentified molecules were named “NI1–NI52”. The PLSDA score plot is represented in Figure 1a for the first two components, with a covariance of 24.7%, while Figure 1b represents the ranking of the first 15 molecules which may explain the discrimination between groups C and P. The statistical significance of the discrimination was certified by the cross-validation algorithm which showed a high accuracy and R2 value (close to 1) and significant, high Q2 values (>0.8), indicating very good validation and predictability for this model (Figure S1).

#### 3.1.2. Significant Metabolites Based on Multivariate Analysis

From the initial number of 123 common metabolites in both groups, based on significant t- and *p*-values < 0.05, 69 molecules were identified based on the parental ion value (m/z), as presented in Table S1 including their Pubchem codes (https://pubchem.ncbi.nlm.nih.gov/; accessed on 20 March 2021), as possible biomarkers involved in the metabolic pathways of acute pancreatitis.

Table 3 includes the parameters that explain the discrimination between these groups (fold change, log_2_FC, *p*-values) and the significance (decrease or increase in the P group compared to the C group).

#### 3.1.3. Random Forest and Biomarker Analysis

**Figure 2:**
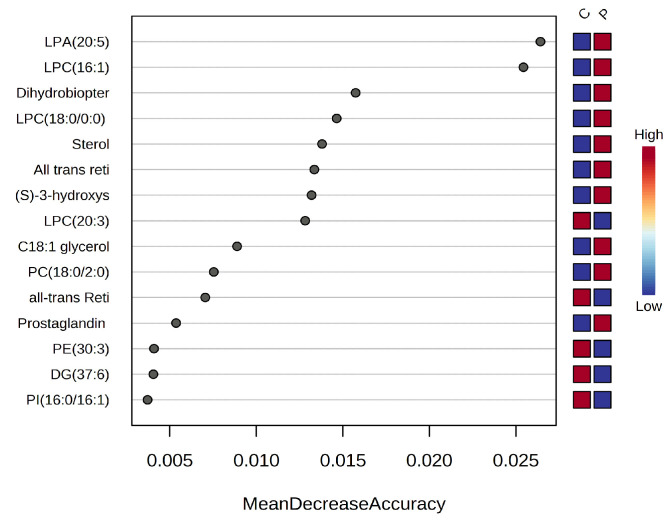
The ranking of the first 15 molecules, according to RF analysis, as predictive biomarkers for acute pancreatitis (group P) vs. controls (group C).

Besides the information presented above, the random forest (TF) algorithm was able to indicate more accurately the predictive value (as potential biomarkers) of the metabolites that differentiated the C and P groups. Figure 2 presents the m/z values of the first 15 molecules to be considered as predictive by the RF analysis, according to the mean decrease accuracy (MDA) values higher than 0.002.

According to this analysis, increases in group P metabolites such as LPA (20:5), LPC (16:1), dihydrobiopterin, LPC (18:0), sterol, all-trans-retinol, and C18:1 glycerol were noticed. Meanwhile, metabolites like LPC (20:3), PE (30:3), and DG37:6 decreased in the P group.

Biomarker analysis was also conducted, building the receiver operating characteristic (ROC) curve, as a useful tool to evaluate the sensitivity vs. specificity of each molecule considered as a potential biomarker. Table 4 shows the m/z values and putative identification of the first 19 molecules with AUROC values > 0.8, *p*-values < 0.00003, and log_2_FC values for each molecule identified, indicating their increase or decrease in the P vs. C group (negative log_2_FC indicates increases in metabolite levels in the P group while positive values indicate decreases in P vs. C group).

The graphical representation of the first 10 molecules to be considered as putative biomarkers of acute pancreatitis is presented in Supplementary File Figure S2.

The most significant metabolites were LPC (20:3), all-trans-retinyl oleate, and LPE (P-16:0/0:0), which decreased in the P group, and LPC (16:1) and LPA (20:5) with increased levels in the P group. Also, dihydrobiopterin showed increases in the P group. These results are in good agreement with the RF analysis and offer a better image of the metabolic disturbances in pancreatitis.

**Figure 3:**
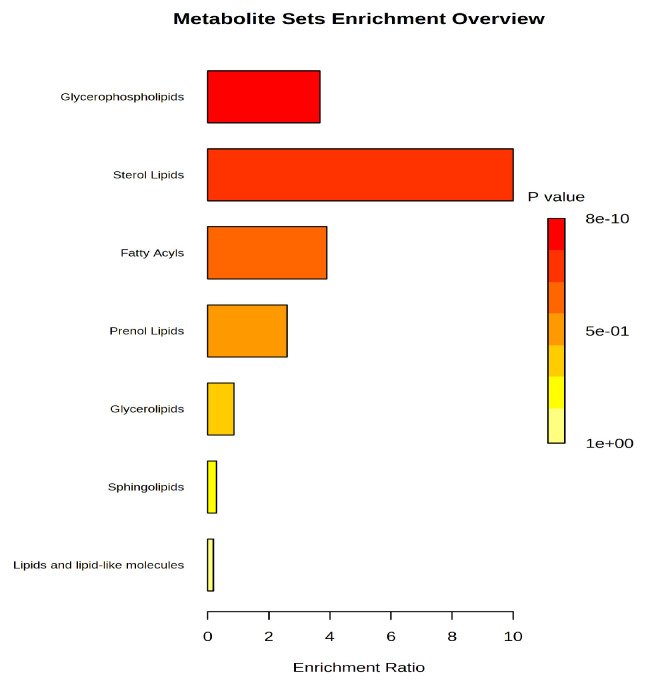
The graph of enrichment analysis, showing the main classes of lipids that may be considered as putative biomarkers for acute pancreatitis.

The enrichment and pathway analysis complemented the above-mentioned results and showed the sub-classes of molecules that are relevant in this study. Figure 3 includes the graph of this analysis.

Therefore, sterol lipids and glycerophospholipids (mainly lysoderivatives) were mostly affected by pancreatitis, as well as fatty acids and prenol lipids.

### 3.2. Multivariate Analysis to Discriminate between Biliary (BAP) and Ethanolic (AAP) Acute Pancreatitis

#### 3.2.1. Discrimination Analysis by PLSDA and VIP Scores

**Figure 4:**
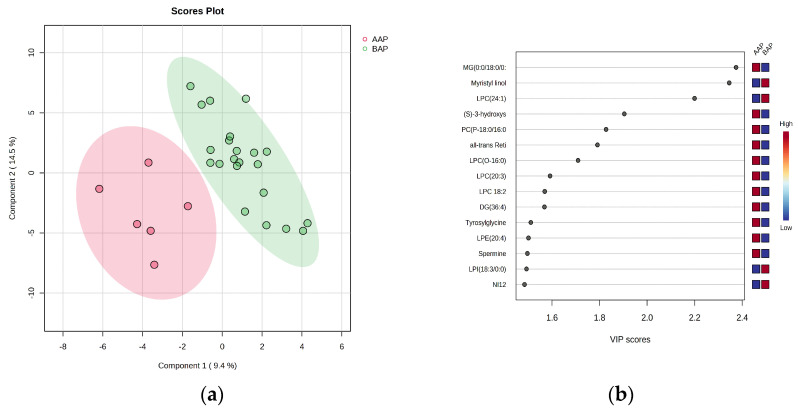
The PLSDA score plot (**a**) and the VIP scores (**b**) higher than 1 for discriminating between BAP and AAP groups. (**a**) PLSDA score plot, showing the discrimination between BAP and AAP groups. (**b**) Ranking of VIP scores for the first 15 molecules which may explain the discrimination between groups BAP and AAP.

The multivariate analysis using PLSDA and VIP scores higher than 1 (presented in Figure 4) shows good discrimination (covariance of 23.9% for the first two components), but due to the small size of the AAP group, the cross-validation algorithm showed lower accuracy (0.65), an R2 value of 0.45, and nonsignificant Q2 values for the first two components, indicating a lower predictability of this model compared to the P vs. C group comparison (see Section 3.2).

This model had lower R and Q values, showing nonsignificant differences between these groups. The VIP scores higher than 1 (Figure 4b) show the ranking of the first 15 molecules. According to the *t*-test, only seven molecules showed *p*-values < 0.1, namely MG (0:0/18:0/0:0), myristyl linolenate, LPC (24:1), (S)-3-hydroxystearic acid, PC (P-18:0/16:0), all-trans-retinyl oleate, and LPC (O-16:0)

#### 3.2.2. Biomarker Analysis

Table 5 shows the metabolites with the highest AUC values (>0.72) and *p*-values < 0.1.

**Figure 5:**
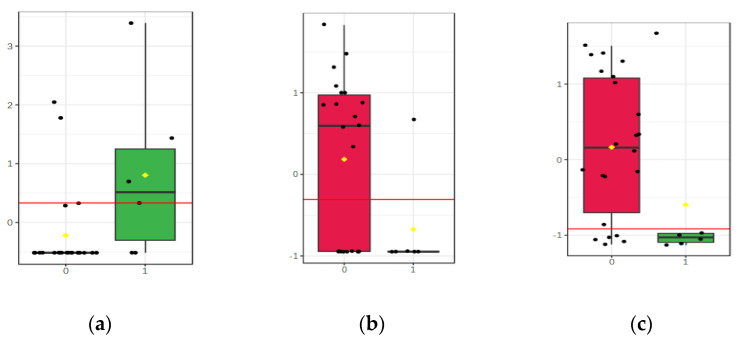
(**a**) Comparative levels of MG (0:0/18:0/0:0) in group 1 (AAP) vs. group 0 (BAP). (**b**) Comparative levels of lauryl stearate in group 1 (AAP) vs. group 0 (BAP). (**c**) Comparative levels of myristyl linolenate in group 1 (AAP) vs. group 0 (BAP).

Figure 5a–c represent, in arbitrary units, the significant differences between the levels of these three molecules.

According to these data, the monoglyceride of stearic acid and esters like lauryl stearate and myristyl linoleate may be considered as putative biomarkers of the differentiation between groups BAP and AAP.

## 4. Discussion

The pathophysiology of acute pancreatitis has been a topic of extensive research over the past decade. We conducted a thorough metabolomic analysis, illuminating significant changes in the lipidomic profile associated with acute pancreatitis. The findings significantly contribute to the understanding of this disease, particularly the profound alterations observed in a wide array of lipids and metabolites implicated in its progression and inflammatory response.

Our study focused on a spectrum of metabolites, namely glycerophospholipids, fatty acyls, glycerolipids, sterol lipids, sphingolipids, prenol lipids, and others. Of these, glycerophospholipids such as LPC (20:3), LPC (16:1/0:0), and LPC (18:0/0:0) played a prominent role, correlating with prior findings regarding their association with inflammation and tissue damage [14]. This pattern suggests that disruptions in glycerophospholipid metabolism may underpin inflammation in acute pancreatitis.

Fatty acyls, including all-trans-retinyl oleate and (S)-3-hydroxystearic acid, were found in elevated levels. While the precise role of these lipids in acute pancreatitis remains ambiguous, they are suspected to play a crucial role in inflammatory responses [16]. Particularly, retinoids such as all-trans-retinyl oleate have known immunomodulatory functions [22], and hydroxystearic acids have recently been linked to lipid-induced inflammation [23].

This research also detected significant alterations in glycerolipids and sterol lipids, aligning with previous research suggesting a disrupted glycerolipid metabolism in pancreatitis [24]. Key glycerolipids such as DG (20:4/20:5/0:0) [iso2] and TG (49:3) are integral to lipid signaling, energy storage, and membrane trafficking, and their aberrations may contribute to pancreatitis progression [25].

A noteworthy change was also observed in sterol lipids, including metabolites like 18:2 cholesterol ester and 1a,25-dihydroxypentyl cholecalciferol. Considering the crucial functions of sterol lipids in maintaining membrane integrity and regulating immune responses, such alterations could signify an adaptive response to inflammation and tissue injury in acute pancreatitis [26,27,28].

Sphingolipids, such as Cer (d18:0/15:0) and Cer (t18:0/19:0(2OH)), also were found as biomarkers of AP, aligning with increasing evidence of their involvement in inflammatory and apoptotic pathways [24]. In the context of acute pancreatitis, disruptions in sphingolipid metabolism may exacerbate the inflammatory response and tissue damage and potentially lead to organ failure [29].

Further, elevated levels of prostaglandin E2, a potent inflammatory mediator, were detected. Though the role of prenol lipids in acute pancreatitis needs further investigation, this rise may indicate the severe inflammation characteristic of this disease. This study also noted significant changes in several other metabolites, including 9-Hexadecenoylcholine and β-Neuraminic acid, suggesting their potential as markers of acute pancreatitis [30].

Comparatively, in two metabolomics studies by Huang et al. and Xiao et al., a consensus was observed in terms of elevated levels of glycerophospholipids and fatty acyls in acute pancreatitis [10,15]. In Huang’s study, multiple lipids, including glycerophospholipids, were identified as crucial contributors to the metabolic alterations of acute pancreatitis. The researchers reported these lipid perturbations might be associated with an exacerbated inflammatory response and tissue damage in acute pancreatitis, corroborating our findings [15].

Notably, the global metabolic phenotyping of acute pancreatitis conducted by Villasenor et al. presented a broad array of metabolites implicated in the pathogenesis of acute pancreatitis. The study particularly highlighted the elevation of lipid metabolites in the patient group. While the authors did not specify the exact lipid species, the overarching theme of lipid perturbations in acute pancreatitis seems to echo the lipid-centric findings from our search [2].

All these findings can lead to a deeper understanding of the lipid-mediated inflammation and tissue damage seen in acute pancreatitis. Lipid dysregulation, as suggested by these studies, potentially contributes to the hyper-inflammatory state observed in acute pancreatitis. The highlighted glycerophospholipids, for instance, play a crucial role in the formation and functionality of cell membranes and can trigger inflammatory pathways when dysregulated [31]. Additionally, fatty acyls, like retinyl oleate, modulate inflammation and immunity, which are crucial aspects of acute pancreatitis pathophysiology [32].

The highlighted sterol lipids, particularly the cholesterol esters, can contribute to cell membrane integrity, and their alterations might relate to cellular damage observed in acute pancreatitis. Our study proposition of glycerolipids, such as DG (17:1/20:5/0:0) [iso2] and TG (57:3), also reinforces the concept of lipid dysregulation. Dihydrobiopterin, a pteridine metabolite, is known for its involvement in nitric oxide synthesis, which can contribute to the inflammatory process [33].

Lou et al. also conducted a thorough investigation of the metabolomic changes in acute pancreatitis but focused on plasma extracellular vesicles. Extracellular vesicles (EVs) play crucial roles in cellular communication and pathophysiological processes, including inflammation and immune responses. The researchers’ quantitative metabolic analysis of plasma EVs provides a unique perspective into the diagnostic potential of these vesicles in severe acute pancreatitis. Comparing the specific metabolites identified in the two studies, both show profound alterations in glycerophospholipids. Similarly, fatty acyls such as all-trans-retinyl oleate and (S)-3-hydroxystearic acid were found in both studies. The consensus on these metabolites underlines the crucial part they may play in the inflammatory response and disease progression. The analyses of glycerolipids by both research groups also mirror each other, further supporting their potential significance in acute pancreatitis [32]. However, some differences in the findings of the two research groups exist. Our study includes a wider range of lipid classes such as sterol lipids and sphingolipids, while Lou et al.’s analysis, focused on plasma EVs, might not cover the same breadth [34].

The identification of metabolites capable of differentiating between the various causes of acute pancreatitis is a targeted development in the personalization of diagnosis and treatment for this condition. Huang et al.’s study is the only one we found that concentrates on using metabolomics to distinguish between the etiologies of acute pancreatitis, yet their findings offer different perspectives [15].

The metabolites proposed by our study span across various lipid classes, such as glycerophospholipids, glycerolipids, and fatty acyls. Notable among these are N-palmitoleyl glutamine, lauryl stearate, myristyl linolenate, 9-Hexadecenoylcholine, LPC (20:3), palmitoylcholine, LPC (18:2), PE (14:1/14:0), MG (20:0/0:0/0:0), and oleoyl palmitoyl glycerol DG (18:1/16:0/0:0). These metabolites were found to have potential discriminative ability in distinguishing biliary and ethanolic acute pancreatitis.

On the other hand, Huang et al. focused on the lipidomic changes related to three different types of acute pancreatitis, including alcoholic, biliary, and hypertriglyceridemic. However, the specific metabolites implicated in their study are not mentioned for a direct comparison. It is worth noting that their findings underscored the importance of lipidomic alterations in acute pancreatitis of different etiologies.

We found changes in glycerolipids, namely oleoyl palmitoyl glycerol DG (18:1/16:0/0:0) and MG (20:0/0:0/0:0), emphasizing the potential dysregulation of energy metabolism in different etiologies of acute pancreatitis, but the relatively low count of AAP patients in our study hampered our statistical analysis.

In summary, both studies affirm the role of lipidomic profiling in the investigation of acute pancreatitis etiologies. While direct comparison is limited due to the unavailability of specific metabolites identified by Huang et al., it is evident that the metabolomic approach taken by both studies holds great promise for delineating the heterogeneity of acute pancreatitis etiologies.

Despite this study offering a snapshot of metabolite alterations in acute pancreatitis, it is crucial to validate these findings in larger cohorts and over the disease’s course. Future investigations should examine if these metabolic changes are causative or consequential, exploring their roles in disease pathogenesis and their potential as biomarkers for disease diagnosis, prognosis, or therapeutic response [35].

Moreover, studies delving into the mechanistic pathways of these metabolites might unveil novel therapeutic strategies. For instance, targeting specific metabolites might help mitigate the inflammatory response and tissue injury in acute pancreatitis [16,36,37]. This study significantly adds to our knowledge of the connection between metabolic changes and acute pancreatitis, paving the way for a nuanced, multi-targeted approach to managing this complex disease. But, as the several studies published until now on this subject had different ways of reporting their findings, systematization is required; the situation right now looks similar to the Renaissance geographic discoveries—a multitude of possible findings, a multitude of ways to find them, and, above all, a lack of real knowledge for interpreting these findings.

Our comprehensive metabolic profiling combined with those of other researchers provides novel insights into the pathophysiology of acute pancreatitis. The distinct lipid perturbations highlight the potential role of lipid-mediated mechanisms in acute pancreatitis and propose potential diagnostic markers. However, further studies are needed to validate these findings and to explore the exact mechanisms by which these lipids contribute to acute pancreatitis pathophysiology in larger, more diverse cohorts of patients; one source of potential bias in our study is the size of our study groups and the relatively low number of severe AP and AAP patients.

The disparity in the group sizes could introduce potential biases in the statistical analysis and may influence the power of this study.

Furthermore, although the control group was matched for age and sex distribution, using hospital employees as controls introduces an inherent selection bias. Hospital employees may have different lifestyles, dietary habits, or other unmeasured confounders, which might influence the metabolic profile, compared to the general population. Such biases could affect the generalizability of the identified metabolites as potential biomarkers for acute pancreatitis.

It is also noteworthy that while matching for age and sex is crucial, other potential confounders, such as underlying comorbidities, medication usage, and genetic predispositions, were not accounted for. These factors might play a role in metabolic variations and could mask or exaggerate the true differences solely attributed to acute pancreatitis.

## 5. Conclusions

This lipidomic analysis provides an introduction to the metabolic landscape of acute pancreatitis, revealing changes in multiple lipid classes and metabolites. Future research could add and discover new diagnostic biomarkers and therapeutic strategies, enhancing the management of acute pancreatitis.

## Figures and Tables

**Table 1:** Characteristics of the patients with PA enrolled in this study compared to controls.

Characteristics	P Group	C Group	*p*-Value
Age (mean ± SD) years	57 ± 16 years	54 ± 6 years	0.3
Male sex (*n*/total number) %	(18/34) 52%	(14/26) 53%	0.8
BMI (*n*/total number) %			
<18.5	(0/34) 0%	(0/26) 0%	
18.5–24.9	(9/34) 26.5%	(8/26) 30.8%	0.9
25.0–29.9	(11/34) 32.3%	(9/26) 34.6%	0.9
>30.0	(14/34) 41.2%	(9/26) 34.6%	0.7
Smokers (*n*/total number) %	(6/34) 17%	(7/26) 26%	0.5
Alcohol consumption (*n*/total number) %			
Normal consumption	(26/34) 76.5%	(20/26) 76.9%	0.7
Alcohol misuse	(3/34) 8.8%	(6/26) 23.1%	0.2
Binge drinking	(5/34) 14.7%	(0/26) 0%	0.1

**Table 2:** Laboratory data of patients with AP and severity prediction scores.

Laboratory Data	BAP Mean ± SD	AAP Mean ± SD	*p*
Leucocyte number × 10^6^/L	14.990 ± 6.376	2.0191 ± 9.672	0.113
Thrombocyte number × 10^6^/L	252.676 ± 104.403	254.385 ± 94.692	0.969
Hemoglobin g/dL	14 ± 1.6	17 ± 4.9	0.007
Hematocrit %	41.5143 ± 5.0112	48.4857 ± 5.4520	0.004
Glucose mg/dL	140 ± 51	164 ± 51	0.297
AST U/L	188 ± 161	106 ± 88	0.214
ALT U/L	198 ± 140	91 ± 124	0.086
Total bilirubin mg/dL	3 ± 1.9	1.3 ± 0.6	0.030
GGT U/L	543 ± 594	208 ± 254	0.164
Alkaline phosphatase U/L	228 ± 343	77 ± 15	0.260
Creatinine admission mg/dL	1.09 ± 1.5	0.75 ± 0.12	0.583
Creatinine 48 h mg/dL	0.84 ± 1.09	0.61 ± 0.15	0.597
Blood urea nitrogen admission mg/dL	47 ± 30	36 ± 11.5	0.362
Blood urea nitrogen 48 h mg/dL	32 ± 15	37 ± 24	0.492
CRP 48 h mg/dL	101 ± 103	228 ± 162	0.022
Calcium level 48 h mg/dL	7.4 ± 3.1	7.8 ± 1.1	0.750
BISAP score at admission	1.9 ± 0.8	1.8 ± 0.8	0.804
Ranson score admission	1.6 ± 1.3	1.7 ± 1.3	0.876
Ranson score 48 h	2.9 ± 2.3	3.1 ± 2.2	0.813

AST = aspartate aminotransferase; ALT = alanine transaminase; GGT = gamma-glutamyl transferase; CRP = C-reactive protein; BISAP = Bedside Index Score for Acute Pancreatitis; BAP = biliary acute pancreatitis; AAP = alcoholic acute pancreatitis.

**Table 3:** Fold change (FC), log_2_FC, and *p*-values for the molecules selected as responsible for the discrimination between groups C and P. Column 4 shows the increase or decrease in these molecules in group P vs. C.

Molecule	Fold Change	log_2_FC	*p*-Value	Significance
LPC (20:3)	3.203	1.68	2.86 × 10^−9^	P < C
Dihydrobiopterin	0.049	−4.36	3.83 × 10^−8^	P > C
LPA (20:5)	0.085	−3.56	6.84 × 10^−8^	P > C
LPC (16:1)	0.124	−3.01	1.45 × 10^−7^	P > C
LPC (18:0/0:0)	0.221	−2.18	4.10 × 10^−7^	P > C
all-trans-Retinyl oleate	3.315	1.73	9.94 × 10^−7^	P < C
(S)-3-hydroxystearic acid	0.219	−2.19	3.00 × 10^−6^	P > C
DG (37:6)	0.675	1.55	3.03 × 10^−6^	P < C
LPE (P-16:0/0:0)	0.457	1.67	2.27 × 10^−5^	P < C
PE (30:3)	0.747	1.76	3.38 × 10^−5^	P < C
Stearyl linolenate	0.764	1.86	3.90 × 10^−5^	P < C
DG (40:9)	0.578	1.77	5.37 × 10^−5^	P < C
TG (57:3)	0.489	1.88	5.60 × 10^−5^	P < C
20:1 Cholesterol ester	0.387	1.93	8.01 × 10^−5^	P < C

**Table 4:** The AUROC, *p*-values, log_2_FC, and identification of the first 19 molecules considered as potential biomarkers. Negative log_2_FC indicates increases in metabolite levels in P group while positive values indicate decreases in P vs. C group.

Molecules	AUROC	*p*-Value	log_2_FC
LPC (20:3)	0.900	4.56 × 10^−11^	2.010
LPC (16:1)	0.890	1.98 × 10^−8^	−2.502
all-trans-Retinyl oleate	0.890	2.70 × 10^−8^	1.951
LPE (P-16:0/0:0)	0.869	2.27 × 10^−7^	1.040
LPA (20:5)	0.861	1.39 × 10^−6^	−3.185
NI44	0.860	2.13 × 10^−6^	0.657
Dihydrobiopterin	0.844	6.57 × 10^−7^	−3.972
LPC (O-16:0)	0.831	5.15 × 10^−5^	0.889
Cholesterol ester 18:2	0.829	7.59 × 10^−6^	0.655
PE (30:3)	0.822	1.28 × 10^−5^	1.129
DG (37:6)	0.822	6.66 × 10^−6^	0.870
20:1 Cholesterol ester	0.819	8.18 × 10^−6^	0.665
N-stearoyl phenylalanine	0.811	4.92 × 10^−5^	0.987
TG (57:3)	0.810	2.94 × 10^−5^	0.812
Vitamin D2	0.809	2.18 × 10^−5^	0.665
Dihydroxypentyl cholecalciferol	0.807	4.02 × 10^−5^	1.117
PG (12:0/12:0)	0.807	9.07 × 10^−5^	1.044
PG (32:3)	0.802	2.62 × 10^−5^	0.695
PI (16:0/16:1)	0.802	1.05 × 10^−4^	0.789

**Table 5:** The AUROC, *p*-values, and log_2_FC for the 3 molecules differentiating BAP (code 0) and AAP (code 1).

Molecule	AUROC	*p*-Value	log_2_FC
MG (0:0/18:0/0:0)	0.758	0.023	−1.616
Lauryl stearate	0.739	0.061	1.295
Myristyl linolenate	0.727	0.099	1.086

## Data Availability

Data available on request.

## Supplementary Materials

The following supporting information can be downloaded at: https://www.mdpi.com/article/10.3390/biom13101558/s1, Table S1: Identification of 69 molecules based on the parental ion value (m/z) and the Pubchem codes of each molecule (https://pubchem.ncbi.nlm.nih.gov/; accessed on 22 March 2021). Figure S1: Cross-validation graph, according to PLSDA analysis. Figure S2: Representation of the variation in peak intensity for the first 9 molecules from groups C and P, which were selected by biomarker analysis, having AUROC values over 0.8.
