# Supplementary material for: Unraveling the Metabolic Changes in Acute Pancreatitis: A Metabolomics-Based Approach for Etiological Differentiation and Acute Biomarker Discovery
Source: Biomolecules. 2023 Oct 22;13(10):1558. doi: 10.3390/biom13101558 (PMC10605849; doi:10.3390/biom13101558)
Supplement: Supplementary file 1 [file biomolecules-13-01558-s001.zip › Figures and supplmentary files/biomolecules-2583224-supplementary files.pdf]

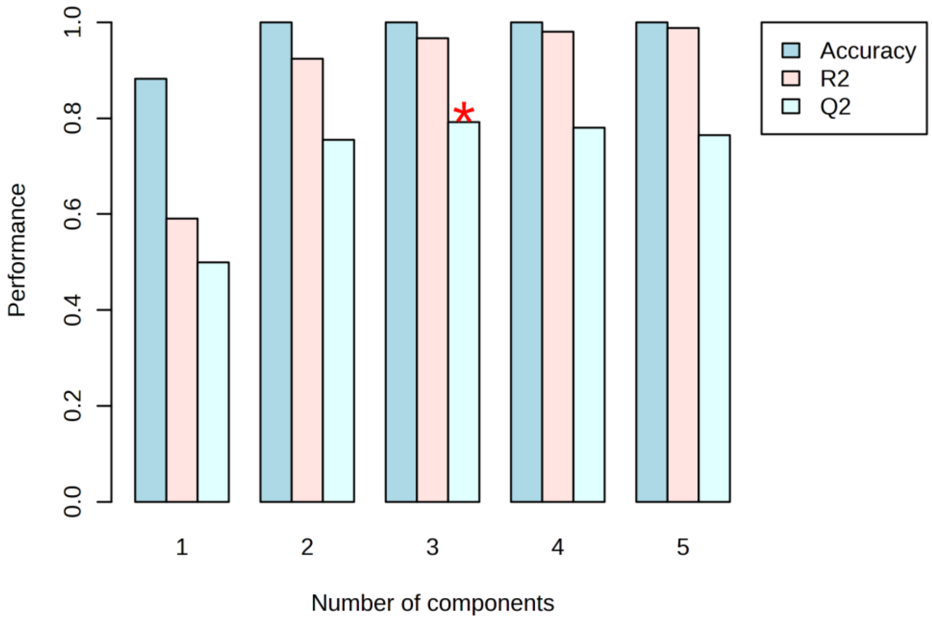

Figure S1. Cross-validation graph, according to PLSDA analysis.

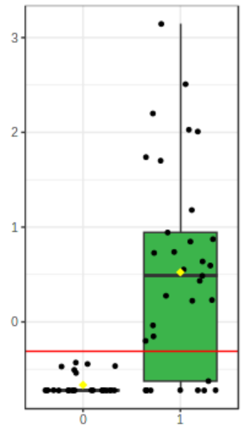

Dihydrobiopterin

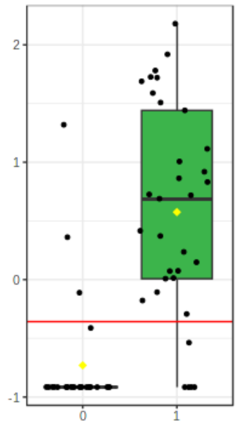

LPC(16:1)

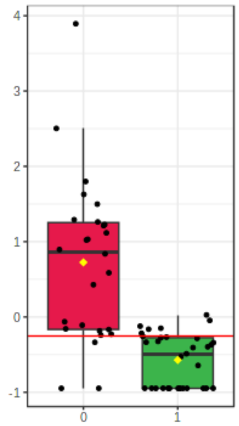

all-trans Retinyl oleate

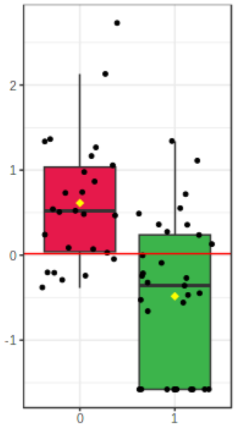

DG(37:6)

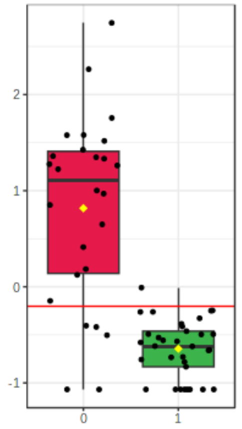

LPC(20:3)

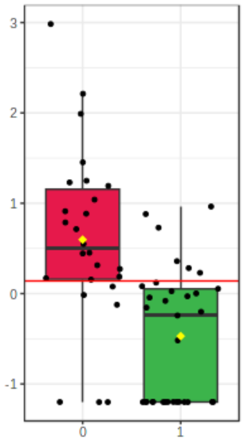

PE(30:3)

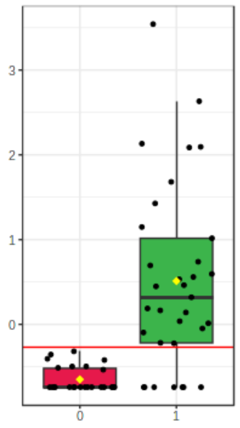

LPA(20:5)

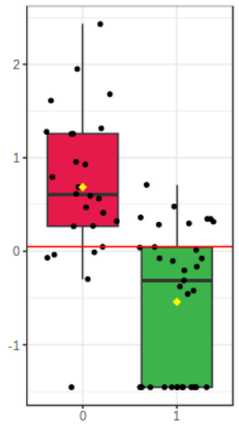

LPE(P-16:0/0:0)

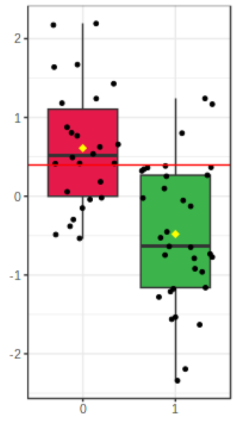

Cholesterol ester 18:2

Figure S2. Representation of the variation in peak intensity for the first 9 molecules from groups C and P, which were selected by biomarker analysis, having AUROC values over 0.8.

Table S1. Identification of 69 molecules based on the parental ion value (m/z) and the Pubchem codes of each molecule (https://pubchem.ncbi.nlm.nih.gov/; accessed on 22 March 2021).

m/z	Identification	Pubchem Code
185.1073	Phosphorylcholine	1014
203.0441	Spermine	1103
239.1652	Tyrosylglycine	29105
240.0977	Dihydrobiopterin	119055
245.0696	N-acetyl spermine	916
249.1503	Sterol	60512
268.0919	beta-Neuraminic acid	513472
301.1313	(S)-3-hydroxystearic acid	54199934
341.2894	9-Hexadecenoylcholine	22155839
353.2567	Prostaglandin E2	5283116
359.3060	MG(0:0/18:0/0:0)	79075
383.1485	LPA(14:0)	71296162
387.2386	MG(20:0)	3246944
391.1525	Homolithocholic acid	15739299
397.2852	Vitamin D2	900
419.1513	1,25-dihydroxy-3-thiavitamin D3	9547302
419.2660	7α,25-dihydroxycholesterol	11954197
423.2349	C18:1 glycerol-3-phosphate	52929771
432.7781	N-stearoyl phenylalanine	6710071
438.3683	LPE(P-16:0/0:0)	42607469
443.3225	Ascorbyl sterate	54725318
453.3310	Lauryl stearate	79186
456.3388	Arachidyl carnitine	6460
457.2116	LPA(20:5)	52929765
475.3127	Myristyl linolenate	56935934
482.3930	LPC(O-16:0)	162126
487.3476	1a,25-Dihydroxypentyl cholecalciferol	9547624
493.2920	1a,25-dihydroxy-11a-phenylcholecalciferol	9547632
494.2988	LPC(16:1)	24779461
502.3515	LPE(20:4)	53480936
513.1999	LPG(18:0)	42607484
518.3038	LPC 18:3	24779469
520.3266	LPC 18:2	11005824

526.4191	Cer(d18:0/15:0)	52931114
531.3729	Stearyl linolenate	6436477
537.2816	Stearyl stearate	17720
539.2975	PG(20:1/0:0)	52927446
542.3086	LPC(20:5)	11757087
544.3266	LPC (20:4)	53480469
546.3747	LPC(20:3)	53480467
546.4428	LPC(18:0/0:0)	497299
551.3141	all-trans Retinyl oleate	11699609
551.3483	*All trans* retinyl oleate	11699609
566.3146	PC(18:0/2:0)	135340
570.4221	DG(16:1/0:0/16:1) (d5)	9543982
575.3987	DG(13:0/20:4(5Z,8Z,11Z,14Z)/0:0)	56936335
579.2820	PA(O-16:0/12:0)	52929565
595.2545	LPI(18:3/0:0)	52928611
595.3682	DG(18:1/16:0/0:0)	5283471
606.319	LPC(24:1)	10406
611.2220	PG(12:0/12:0)	24779549
614.4691	Cer(t18:0/19:0(2OH))	91820041
617.2373	DG(36:4)	07248
627.4187	DG(37:6)	9543769
634.4373	LPC(26:1)	29220
649.4001	Cholesterol ester 18:2	5287939
650.3474	PC(26:0)	24778800
658.4962	PE(30:3)	9543978
663.4496	DG(40:7)	9543882
665.4367	DG(40:9)	9543872
679.4958	20:1 Cholesterol ester	16061337
717.4508	PG(32:3)	52926633
746.5462	PC(P-18:0/16:0)	53480705
763.4447	PG(O-18:0/18:1)	52927275
793.3259	PG(38:7)	52926625
798.5320	PC(P-18:0/20:2)	53480711
809.3022	PI(16:0/16:1)	52928334
815.3178	TG (49:3)	131759279
927.6434	TG(57:3)	56937912
