# Supplementary material for: Unraveling the Metabolic Changes in Acute Pancreatitis: A Metabolomics-Based Approach for Etiological Differentiation and Acute Biomarker Discovery
Source: Biomolecules. 2023 Oct 22;13(10):1558. doi: 10.3390/biom13101558 (PMC10605849; doi:10.3390/biom13101558)
Supplement: Supplementary file 1 [file biomolecules-13-01558-s001.zip › Figures and supplmentary files/matrix final cu ident.pdf]

	P1	P2	P3	P4	P5	P6	P7	P9
	BAP	BAP	BAP	BAP	BAP	BAP	BAP	BAP
Phosphoryl	4007	4084	4816		5329	4208	4644	
NI1	6395	4178	4993	8463	6132	7400	9694	8860
Spermine	5788	3693	9775	5981	6729	5684	6174	
NI2	8199	7514	7506	9469		4735	4580	
NI3	8612	5907	7653	10599	9604	8764	9980	8566
NI4		2183						
Tyrosylglyc	3797	3094		3297				
Dihydrobiopterin				18246	15450	15561	16514	8268
N-acetyl sp	8599	9367	9017	12575	11520	10640		9937
Sterol				15923	14142	16593	12638	7007
b-Neurami	8336	7755	9352	7447	6567	5942	9840	5565
NI5	3917	5931	3175	6422	5260		2099	4546
NI6	13737	5820		12863	6750	6087	11486	4674
NI7	4426	5828	6687	7559	4725	5638	4186	
(S)-3-hydro	3218	3704		7376	5825	7763	7015	
NI8	8362	5734	6686	8846	3333	6522		5342
NI9	2029	2220	1797	2559		1881	2284	
NI10		2656		2317	1798		2418	
9-Hexadecenoylcholin		5068		6828	4938	3744	4685	4062
Prostaglandin E2			6060		10305	2652	6471	4669
NI11	2471		2124		2128		2681	
NI12	13693	8373	11407	15837	13034	12276	15245	11811
MG(0:0/18:0/0:0)								
LPA(14:0)	1939		2378	3475	2791		2708	2286
MG(20:0)	16803	12185	15845	20835	17362	17433	20673	17718
NI13				2259		2323	1903	
NI14	2386					1867	2549	
Homolithocholic acid							3254	
NI15			2261	4321	2943	2960	2361	2692
NI16				2125	2084		1934	
NI17			2723		4404			
Vitamin D2	6882	5688	6277	7839	7547		7622	7987
1,25-dihydroxy-3-thiav		3897	4209	5557	5279	4463	3646	3565
NI18			1642		1847	2317		
7?,25-dihy	15519	9805	12695	17977	14626	12444	16549	12738
NI19				3569	3233			
C18:1 glycerol-3-phos		3783			1739	9754	2124	
N-stearoyl phenylalani		4480	2831		3260		3351	3963
LPE(P-16:0,	3488	4674		3734	3426			4286
Ascorbyl st	257180	224253	276991	329628	327396	314048	338824	7163
Lauryl stea	3873			4235	2896	3336	3822	
NI20								
Arachidyl c	2002		1727	1851	2427	2168	2363	2055
LPA(20:5)				26408	6211	22261	22552	10219
NI21	7780		6255	8379	10554	9245	7365	6476

NI22		8941	5547	7903	8621	8874	7605	8525
NI23				2021				2033
Myristyl lin	151180	12204	155629	171164	16962	146253	177405	163927
LPC(O-16:0	3791	5210	5908	7018	6470	5043	3829	3402
1a,25-Dihydroxypentyl		3691	2952	5548	3872	3418		
NI24	4400	4088	4898	5252	5336	5145	5305	5078
1a,25-dihydroxy-11a-phenylchole(			9794	10916	9357	8359	9391	9828
LPC(16:1)	4662	3431		8765	9878	10759	9411	3908
NI25	18912	43376	49494	73504	102954	119807	72163	86369
LPE(20:4)	9861	9935	3066	9836	8227	5695	8122	9919
NI26		2606		3586				3156
NI27	6791	7564		7094	5626	3965	5314	8236
LPG(18:0)				11874		11080	6203	
NI28		3977	3145	6210	3713	5150	4682	4582
NI29	3071			3501				
LPC 18:3	1635	6164		9130	12127	12289	8540	10763
LPC 18:2	67763	36140	11463	32713	29258	33679	4960	20644
NI30	11346	13312	13663	15027	18964	19431	23369	12229
NI31		12767	12535	15765	13316		10966	12328
Cer(d18:0/	8038	7719	6607	5167	7285	8621	6731	6223
Stearyl lino	4048	3500	2824	3027	3200	4647	3527	2941
Stearyl stea	2669	3342	3050	4400		3978	4034	4101
PG(20:1/0:	3048		3359	3896	3389	3752	3777	3404
LPC(20:5)	8500	5293		3252		3636		3152
LPC (20:4)	29421	23588	19381	34800	21370	19390	4565	7276
LPC(20:3)	3569	3426	3742	5494		5411	3973	3189
LPC(18:0/0:0) [M+Na]		8489	11774	14321	14669	13545	11207	8301
NI32	2257			2780	3013	2637	2373	2547
all-trans Retinyl oleate		4371	5541	8455	6465	6861	6015	4024
NI33	8364	7058	7551	9897	8689		9592	8734
NI34	1487	1749		2457			2604	1653
PC(18:0/2:(	3846	2403		3928				1415
NI35	8652	8152	9438	12413	10381	13486	12309	11512
NI36		4382	4724	5410	5413	2810	2805	5124
DG(16:1/0:	5053	4827	7321	10831		7338	5135	5807
DG(13:0/20:4(5Z,8Z,11Z,14Z)/0:0)			3573	4675	3994	3773		2773
PA(O-16:0/	2478	2441				4589	2568	
NI37		2994	2029	3302			3052	
NI38			1088			2257	1814	
NI39	7691	8161	8277	9697	9166	10513	7945	7154
LPI(18:3/0:0)		3612		4282		5150	3929	4162
DG(18:1/1(	3817	3860	3331	4199		4675	3541	3338
LPC(24:1)	1241	1490		1674		1423	1665	
PG(12:0/12:0)			3464	5205	4119	4772	5051	4654
Cer(t18:0/1	5903	7214	5147	6296	5160	6300	6284	6183
NI40	2520		2682	3851	2866	3360	2389	2411
DG(36:4)	9767	8402	8364	11257	10412	12143	11323	11549

NI41	3133	3920		3197		2092	3227	
DG(37:6)	2520	3063	2460	3896	2705	3470	2909	2652
LPC(26:1)	4019	4450	3709	3582		4728	3863	3799
NI42	1535			2049				
NI43	1267			2807		2731	2273	
NI44		2828			3135		3181	3074
Cholestero	6622	6581	7324	10506	8839	10437	8956	10421
PC(26:0)						1073		
PE(30:3)	3576	3440	2880	5742	5538	5173	3382	
DG(40:7)	2355	2299		3220	2917	3027	1482	
DG(40:9)		7741	6679	9169	7859	7881	7987	8620
NI45			1519				2310	
20:1 Choles	117408	123667	117889	185222	166876	182710	159160	136582
NI46				1390	1635	1631		1756
NI47			1517	1750			2171	1546
NI48	131820	15401	127585	202563	17589	17421	18068	7913
PG(32:3)	3983	4553	4106	6565	5328	6614	4813	4529
PC(P-18:0/16:0)				2016	1855	2372	1887	
NI49	1222	1550	1641	2129		1753		1587
PG(O-18:0/	1536		1458	1752		1550	1609	
PG(38:7)				2281	1892		1609	
PC(P-18:0/	1431	1702		2190	1808	2046		1481
NI50				1840	1538	1444	1126	
PI(16:0/16	2958	3504	3454	5021	4134	4555	3975	4193
TG (49:3)	1573	1780				1955	1748	1729
NI51				1472		1298	1099	
NI52	1081				1186	1481		1139
TG(57:3)	2951	3078	2796	4755	4586	5217	3700	4357

P10	P11	P12	P14	P17	P19	P20	P22	P23
BAP	BAP	BAP	BAP	BAP	BAP	BAP	BAP	BAP
	2851		4586	4173		3378	2944	
8237	8400	7361	8779	4821	6748	6263	5471	4152
11097	8695		6559	2984			5403	
6010	5021	3126	5834	10640	2742	4474		6187
10781	10043	9518	10696	7438	5669	6410	7999	5647
	2867	2382	3218				2225	
7374	6718		10167				3959	
21814	9479		5737	3982	3068		13724	5477
11882	11941	8971	12530	9892	6369	8710		7316
14160	2241	5830	10013	6693		5667		
6579	8951	12628	15567	14580	2865	6120	4724	4604
6274	4272	4589	5326		3196			
	10378	13052	14128	7315			13391	14318
6814	5545	4491	5354		3820	3152	2650	
3223		4569		4437	3824			
6297	3128		8991		6316	4485	6726	6886
	2477						1545	
1205	2066						1528	
3882	4464		3244	2874			3710	
3933		5564	7790		4355	4675		
	2695							
15683	12440	13231	15613	10025	8573	9346	10293	9675
5350							2235	
3014	2727		2594	2121				
23071	23866	18193	24911	15241	9913	12117	17096	11977
2201	2540		2624		1743			
2232	2711	2131	2670	3089		280		
5208								
2936	3475		3316					
2383	2330		2911	1939			1643	
	3951	2595	4149				1727	1602
8252	8172	7449	8648	6732	6097	5169	6468	
4259	6695	4609	6174		3660	3480	2549	3703
1947		1933						
16154	17215	14630	16077	11683	9462	9077	14284	11241
3116	4089	2830	4256	2625	3017		2511	
4632			1441					
4262	4071	2852	3765	3582				
4390	3679	3210	5170	3747	2842	2981	2670	
353203	366664	252554	404480	282241	173023	141291	277487	184068
3843	4957		3566	3367				
		5090				5290		
	2693			2173				
33426	14973	6397	7194	5370	4394		22202	7556
5460	5947	5672	8515	8243	5611	4343	6948	

7105	9525	5593	10566	7865			7523	6535
1937	2709	1385						
169639	15658	119205	26373	13631	101752	72446	9940	102685
5299	3407	3649	4691	4306	4080		4133	
3749	3695	2516	4534	3399	2738	2208	3392	
6306	6227	3886	6638	5272	3388		4094	3367
9150	9282	8746	7638	8570	7230		8822	
2808	3045	6210	1595	6798	5851	6315	3657	4200
61296	53462	49238	66071	48642	16203	34507	22041	8839
14506	9036	7423	10182	12647	4714		2896	5365
4543	4122	2056						
10078	9303	6563	8414	9930		4780	8419	
9741							1393	885
	5656	4351	6482	4156	3236	2302	3590	
	3118		2924	3327				
7561	5942	5331	8024	5687		3500	1735	
26755	10098	5315	30472	36438	18257		40532	
21012	16200	11510	11072	9550	7546	13385	6083	4989
17320	18182	11957	17117		12778		7721	
7378		5822	9250	6090		4712	4769	3688
	4911		4418	3697				2290
5235	5002	3375	5139	4066	2355		3594	
4193	4759		4932	3504			3055	
4043	2094		3257	3693			4227	
7247	29832	16681	35505	30492	23326	9995	23539	5505
5509	4634	3967		3499	2485		2314	3171
13587	15146	12160	17666	9584	8548	9943	8980	
2971	3107	2164	2579				1711	
8082	9086	6297	8488	6285	5493	3963	4350	
11352	9271	8768	10476	7981	7124	6920	7659	6637
2729	2633		2673	2253				
				3898			3250	
16536	14771	10604	17257	10839	7746	7511	11471	6385
6598	5786		5111		3553	4169	2420	5259
7393	3375	4928	7113	5505	4969	4880	5491	
3540	3501		4738	3312		2605	2854	
6889	3913	5299	7725	3804				
2418				2886			1685	
	2900		3034					
7493	6851	7662	11536	3928	7133	5119	3120	5142
	4668	3758	4826	4296			1819	1813
3172	4703	3143	5931		3244			
1971	1969		2254		1125			1076
5362	5774	3626		3641	2920		3207	
7506	7709	5532	9454	4865	4880		4459	3669
3390	3096	2427	3624	2166	3672	2208	2716	2583
11919	11786	9609	12662	10213	8073	6355	8702	

	4270	3414	4651		2185		1048	
4281	4001	3529	4948	2608	2122		2177	
	2554		3400		3701		3217	3535
	2161			1819				
2314	2556		3290	1688	1411	1527		2070
4132	3548		4842				2342	
12087	10059	8560	13486	9417	6047	6256	704	4631
1629								
4255	3305		4071	3672		2099	3942	
3226			3343				1923	
10117	11318	7385	10336	8336	4592		7179	
		1952						
205613	212744	117305	4890	142717	98641	74639	121228	84145
	1329		1896					
2003	1873			1694				
22008	13247	125447	17294	144398	109159	81483	11901	85310
6866	6888	3884	6719	4131	3847	2131	4535	2640
	1678		2438					
1792	2142		1622	1390	1196		1519	
1642	1896				1437		1473	
2575	3015		3288	2051				
1890			2736				1471	
	1911		2340				1126	
5125	5477	3551	5959	4688		3224	3532	1969
2290	2283		2211				1636	
2205	2220		2405	1192			880	
1501	1697	1132	1778	1441				
5040	6604	2752	6778	4419	2917	2335	3671	1762

P25	P29	P30	P33	P34	P8	P15	P18	P21
BAP	BAP	BAP	BAP	BAP	AAP	AAP	AAP	AAP
					5009	3115		
5628	4656			4122	10765	7560	4290	4753
10716	4392				6291	6960	8418	
5286				3450	4321	5178	3896	8826
5550	3509	2261	3192	4686	10964	10263	6207	6422
					3481			
						12126		4701
	7520	6616	5441	7757	13925	8934		3322
8438					9868	11929	7841	8165
	13684	9439		7145	29098	8658		6552
					3380	16571	4165	7281
5776		4582		4939	5092		1739	5815
12676	1586	11960		10415	10679	15268	11053	4629
4354		4721		3962	4804	2668	3623	4907
				3018	4097	5387	3511	4134
4377					9177	9090	5248	7070
						1377		
							1260	
					3771	3579	3314	3344
2480		4638		4843	8494	6912		4635
9405	7659	5708	6215	9008	15563	15412	9382	8832
5924	2146						4610	2244
						2273		
11131	6866	3897	6686	6878	19835	24529	12894	12238
							2280	
1146						2808		
8245			6360				12283	8379
					3424	2407	2359	
					2118		1222	
							3154	3413
	2568				7851	8633	5106	6133
					2825	4493		
10915	7471	8362	6432	9108	15907	17040	10247	9845
					2261	3579		
		5209	3439			3418		
					4424		3322	
					4259	4422	3485	3296
169105	118758	109103	108442	149648	329313	329767	213105	192640
4090	5589		4090	4657				3500
						2386		
	11469	9682	7386	9571	19076	13944		4427
5819					9271	10518	9037	4095

5514		3862		3721	8414	10309	8858	3581
3089	3390		3879			1476	1645	2890
94257	66924	71074	67719	88742	187517	19538	14170	10664
3059				3008		8403	3391	3996
						4961	2958	
2671	2047		2336	3159	5742	5893	4036	4395
					8772	6264		2282
	7163	6502	2417	5728	3649	8266	3447	5829
54529	18057		13755	41741	95532	47371	5370	52731
8517					8960	10370	7261	8349
					2539	3965		
		2809	3609	5046	6914	12329		8000
	10318	6518			23502	4118		
2240					4938	6438	2846	2313
						4515		
5320				4053	11691	6610	1259	5423
20325			4440	14629	17470	38905	22993	40125
6816		9661		4488	15095	12939	4999	7396
		4506		4504	12557	16535	9810	10377
		3734			7151	7174	4806	
					3304	3791		
1627	1398				4446	5133	2580	
2380				1968	3971	5280		2291
						3940		4813
14063	9691	6626		9890	7559	41320	21828	5511
2458		5003		1843	4163	5419	6995	2941
		6068			11110	9371	4881	9550
1310		3618			2411	3371	2125	1844
	2555	3140			6364	8767	2601	5152
6566	3913				9480	11162	7336	7321
							1488	
						4664	2614	
6522	3162	2415		3768	11088	19748	9126	7911
				3229	4444	5613	2854	
3706				3049	5625	8531	5522	4869
					2801	5212		3119
					3878	5218		
						2760		
							1065	
1872		1028	1176	3208	7803	9673	4635	3400
2455						5715		
					3815	5406		
					5232	3711		
3877		2782			6714	8068	4009	
2144				1724	3022	4459	2464	2106
4467					12266	12876	7103	7121

	1229				3558	3790		
					3679	5343	2308	2273
				1833	3782	5364	4536	2377
					1870			
					2266	3089	2107	1306
					3347	6380	1982	
5391	3107	3206	2842	4440	10269	13759	6204	5545
					1095	1851		
					3269	3176	2779	2991
					1778	3379	1785	2244
					6444	9661		
					1856	2080		
64172	37558	44379	41078	57571	136860	235434	111768	90622
					1422			
					1587	2486	1552	
64724	33745	46936	43248	67777	8909	25752	113795	97522
					4607	8277	3476	3615
					1578	1850	1504	
					1728	2792		
					1482	1241		
					2726	3350		
						2272		
					1411	2386	1061	
	1140				4660	6604		2679
					1846	2196		
						2992		
					976	2461	1232	
2055				1789	4559	7181	3246	2137

P24	P27
AAP	AAP
2898	
2626	5397
	18440
7652	
5380	5573
3690	
6918	8315
8039	6548
	18293
7534	2670
3661	5445
6406	
	4880
2425	3227
5553	
3558	
6912	7714
3026	8805
	1331
9261	8987
	2497
1417	
6565	
4783	2996
8548	9392
	1914
2891	
179239	165638
5480	
8582	10694
3887	

3913	
17782	9129
4753	4374
3781	2936
9009	
	9105
30600	45012
3368	9112
6439	6598
	11508
	4504
27938	16104
12151	9286
15099	
5468	4403
3201	
2351	
	1919
4557	
7189	
3952	2157
13075	6751
8193	4347
	5242
	4403
3838	4894
4426	2947
8307	4096
4098	
3320	
5724	4464
2763	1752
9358	5581

3270	
1950	
3249	
7042	6107
6281	
91810	74585
5611	85386
3152	2189
	1458
	952
	1371
3141	
825	
3733	2025
